# Mental Health Practitioners’ and Young People’s Experiences of Talking About Social Media During Mental Health Consultations: Qualitative Focus Group and Interview Study

**DOI:** 10.2196/43115

**Published:** 2023-04-07

**Authors:** Jane Derges, Helen Bould, Rachael Gooberman-Hill, Paul Moran, Myles-Jay Linton, Raphael Rifkin-Zybutz, Lucy Biddle

**Affiliations:** 1 Population Health Sciences Bristol University Medical School Bristol United Kingdom; 2 Centre for Academic Mental Health Bristol University Medical School Bristol United Kingdom; 3 Gloucestershire Health and Care NHS Foundation Trust Child and Adolescent Mental Health Services Gloucester United Kingdom; 4 Medical Research Council Integrative Epidemiology Unit University of Bristol Bristol United Kingdom; 5 Translational Health Sciences Bristol Medical School University of Bristol Bristol United Kingdom; 6 The National Institute for Health Research Applied Research Collaboration West (NIHR ARC West) Biomedical Research Centre University Hospitals Bristol and Weston NHS Foundation Trust Bristol United Kingdom; 7 Biomedical Research Centre University Hospitals Bristol and Weston NHS Foundation Trust Bristol United Kingdom; 8 School of Education University of Bristol Bristol United Kingdom; 9 Bethlem Royal Hospital South London and the Maudsley NHS Foundation Trust Beckenham United Kingdom

**Keywords:** young people, digital technology and social media, mental health consultations, clinician and young people’s experiences

## Abstract

**Background:**

Increasing concerns among mental health care professionals have focused on the impact of young people’s use of digital technology and social media on their mental well-being. It has been recommended that the use of digital technology and social media be routinely explored during mental health clinical consultations with young people. Whether these conversations occur and how they are experienced by both clinicians and young people are currently unknown.

**Objective:**

This study aimed to explore mental health practitioners’ and young people’s experiences of talking about young people’s web-based activities related to their mental health during clinical consultations. Web-based activities include use of social media, websites, and messaging. Our aim was to identify barriers to effective communication and examples of good practice. In particular, we wanted to obtain the views of young people, who are underrepresented in studies, on their social media and digital technology use related to mental health.

**Methods:**

A qualitative study was conducted using focus groups (11 participants across 3 groups) with young people aged 16 to 24 years and interviews (n=8) and focus groups (7 participants across 2 groups) with mental health practitioners in the United Kingdom. Young people had experience of mental health problems and support provided by statutory mental health services or third-sector organizations. Practitioners worked in children and young people’s mental health services, statutory services, or third-sector organizations such as a university counseling service. Thematic analysis was used to analyze the data.

**Results:**

Practitioners and young people agreed that talking about young people’s web-based activities and their impact on mental health is important. Mental health practitioners varied in their confidence in doing this and were keen to have more guidance. Young people said that practitioners seldom asked about their web-based activities, but when asked, they often felt judged or misunderstood. This stopped them from disclosing difficult web-based experiences and precluded useful conversations about web-based safety and how to access appropriate web-based support. Young people supported the idea of guidance or training for practitioners and were enthusiastic about sharing their experiences and being involved in the training or guidance provided to practitioners.

**Conclusions:**

Practitioners would benefit from structured guidance and professional development to enable them to support young people in feeling more willing to disclose and talk about their web-based experiences and their impact on their mental health. This is reflected in practitioners’ desire for guidance to improve their confidence and skills to safely support young people in navigating the challenges of the web-based world. Young people want to feel comfortable discussing their web-based activities during their consultations with mental health practitioners, both in tackling the challenges and using the opportunity to discuss their experiences, gain support, and develop coping strategies related to web-based safety.

## Introduction

### Background

Young people’s use of digital technology and social media is ubiquitous and presents a new set of challenges in relation to web-based safety. Increasing concerns among mental health professionals and members of the public have focused on the impact of digital technology and social media use on young people’s mental well-being [1]. Over 90% of young people in the United Kingdom aged between 16 and 24 years use social media [2]; >90% of children aged 8 to 11 years spend >12 hours a week on the web, and most adolescents aged 12 to 15 years spend at least 20 hours a week on the web [3]. Web-based activities have been associated with poor sleep, anxiety, and depression [4,5], especially among young people who are already vulnerable and whose mental health problems can be amplified by increased time spent on social media [6]. Poor mental health has also been linked to the increased availability and accessibility of distressing images, cyberbullying, web-based sexual exploitation, and web-based gambling [7]. Concerns about the increase in screen time, especially among younger age groups, is reflected in the number of adolescents (5%) showing harmful behaviors such as compulsive checking [3]. Reports have also highlighted the dangers of social media’s influence on younger people in relation to the incitement to self-harm and, in some instances, suicide [8-10]. Teachers report the effects of problematic digital technology use among schoolchildren, leading to increased levels of sleep deprivation affecting concentration and schoolwork as well as behavioral problems linked to cyberbullying [11,12].

There have been growing calls for digital technology and social media companies to improve the safety features of their platforms, such as removing harmful content and increasing site moderation [13]. However, as the Royal College of Psychiatry report on young people’s mental health and technology use also highlights, although it is essential for social media companies to address the issue of web-based risk, it is also important to support young people in engaging more safely with the web-based world and in a way that has a positive impact on their well-being [14]. Often unrecognized is the role social media plays in supporting young people’s mental health by providing information, a sense of community, and emotional support [15-19]. However, the types of social media use are heterogeneous and can depend on whether the young person is an active or passive participant on social media; for example, findings suggest that passively looking at posts and images has a more problematic impact on mental health than active engagement [20]. Digital technology plays a large part in young people’s lives, and its potential influence on their mental health, both positive and harmful, needs to be explored routinely during clinical consultations if young people are to be supported and encouraged to engage safely on the web in relation to their mental health. Potential topics for discussion include types of web-based activities; screen time; web-based gambling; and, more broadly, their experiences and interactions on social media [14].

### Objectives

Our linked quantitative work identified that only 49.4% of practitioners routinely ask young patients about their web-based activity, and of those young people asked, only 36.2% said that it was helpful [21]. We also identified a gap in understanding between practitioners and young people in terms of how much practitioners appreciate how young people engage with the web-based world [21]. We aimed to explore whether, how, and when clinical conversations about web-based activities occur with young people by seeking the experiences of both practitioners and young people. In particular, we sought to understand how such conversations, when they take place, are perceived by young people as their views are often absent from the evidence base. Practitioner perspectives on whether there is a need for guidance or training were also explored.

## Methods

### Overview

We conducted a qualitative study using interviews and focus groups with practitioners and young people. These began by exploring experiences of digital technology more broadly before characterizing experiences of talking about mental health–related web-based activities during consultations. *Web-based activities* are defined here as the use of digital technology to access social media, messaging services including both SMS text messages and web-based messaging apps such as WhatsApp, web-based games, and websites.

### Recruitment and Sample

**Figure 1:**
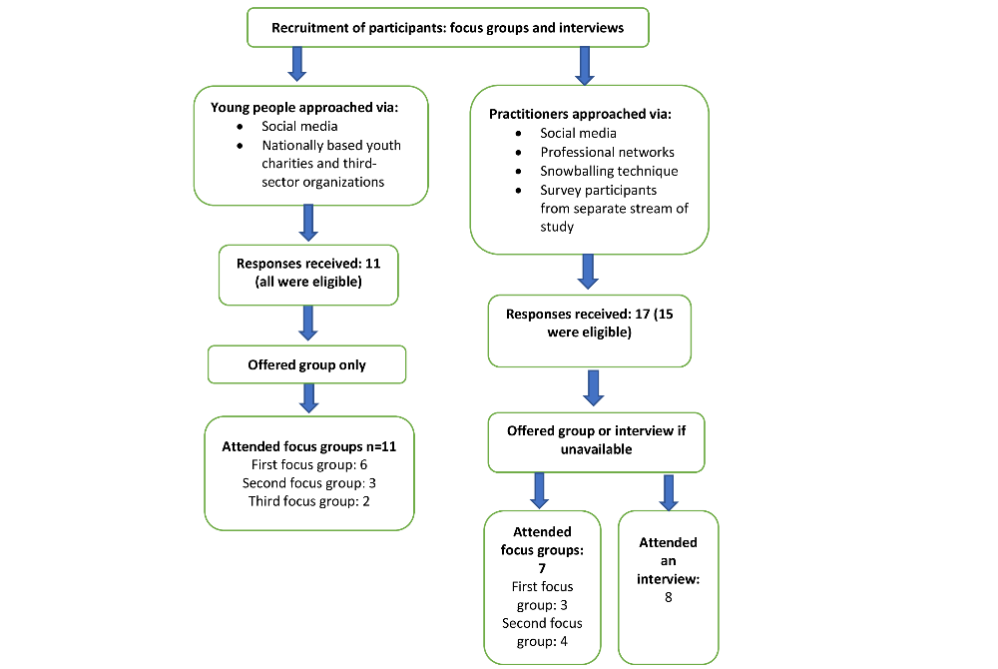
Recruitment and sample.

Recruitment took place in 2020. A multitude of approaches were used to enhance the diversity of the sample obtained, as summarized in Figure 1.

#### Practitioners

Practitioners were recruited by advertising on social media, for example, Twitter; within the professional networks available to the research team; using snowball sampling; and by inviting participants of a web-based survey conducted at an earlier phase of the research who had expressed an interest in continued involvement. Practitioners were eligible to take part if they were qualified professionals currently working in one or more of a range of young people mental health services, including children and young people’s mental health services (CYPMHS), the private sector, student well-being services, or young people third-sector mental health organizations where they provided support to those aged between 16 and 24 years. Unqualified individuals were not eligible to take part, for instance, health care assistants, trainees, and students, as well as those working solely in adult mental health services. Of those who expressed interest in taking part, 2 were not eligible (a health care assistant and a psychology student). There was a wide range of years of practice, which influenced the depth of experience that practitioners brought to the study.

#### Young People

Young people aged 16 to 24 years with lived experience of mental health problems who had previously sought help from mental health services (statutory outpatient services or private or charity sector) and who were active on the web were eligible. Young people were not eligible if they were receiving inpatient care at the time of the study. Participants also had to have sufficient command of the English language to enable them to participate in a web-based conversation and be currently living in the United Kingdom. The study was advertised through social media and the study team’s contacts in third-sector young people mental health organizations. As with the practitioner participants, individuals who had participated in a web-based survey from an earlier phase of the study and expressed an interest in continuing to be involved were contacted (Figure 1). All who expressed an interest in participation were invited to attend a focus group, and of the 11 who responded to the advertisements placed on social media, through third-sector contacts, and the web-based survey, all (100%) were eligible.

### Ethics Approval, Participation, and Informed Consent

Approval from the University of Bristol Faculty of Health Sciences Research Ethics Committee (reference 103102) was obtained in June 2020. Informed consent was obtained through written signed consent and, where applicable, appropriate adult assent. Privacy and confidentiality were ensured throughout; the transcripts were only accessible to the 2 researchers involved in coding and analyzing data (JD and LB, both female with PhDs in the social sciences). All participants’ names were removed from the transcripts and replaced with ID codes. Once data analysis was complete, all audio recordings of the interviews and focus groups were deleted. Each young person participant received a £20 (US $24.32) gift voucher, and each practitioner participant received a £10 (US $12.16) gift voucher.

### Data Collection

Data collection took place between June 2020 and October 2020. Health practitioner participants had the option to take part via one-to-one interviews or focus groups because of organizational challenges of attending a group. Topic guides were used for the focus groups and interviews to elicit and explore similar topic areas but were used flexibly to encourage free discussion. In the practitioner focus groups, we discussed participants’ individual experiences of having conversations with young people about their web-based activities related to their mental health. Additional information was obtained about participants’ knowledge of digital technology and social media to identify any areas of expertise or gaps in knowledge. For the young people focus groups, emphasis was placed on how they used digital technology and social media and their experiences of talking to practitioners about their web-based activities related to their mental health.

Each focus group lasted approximately 1 hour, and the interviews lasted approximately 45 minutes. Focus groups were conducted via a web-based video platform (Zoom Video Communications), and the interviews were conducted through either web-based video or telephone. All participants were audio recorded with their consent. Information sheets were provided to each participant.

### Analysis

Audio recordings were transcribed verbatim and then anonymized and analyzed using the reflexive thematic analysis approach as described by Braun and Clarke [22]. This approach was used to assist in understanding the experiences, thoughts, and behaviors of practitioners and young people in talking about young people’s social media use during mental health consultations. This topic is relatively unknown, and because of the complex nature of the data set, the 2 participant groups (clinicians and young people), and the 2 methods of inquiry (focus groups and interviews), we decided that a more reflexive approach would allow us to look for patterns of meaning across all the data to support a process of ongoing interpretation that would help develop a richer analysis [22-24].

Transcripts of the focus groups and a selection of interviews with both practitioners and young people were independently read through by the researchers (JD and LB) to begin identifying possible meanings within the data before inductive coding. Both sets of codes were then compared for quality assurance purposes before being synthesized, and a full list of codes was derived for each participant group.

Coding helped organize and initiate the process of identifying connections across the data, including any inconsistencies, as well as highlighting any questions for further exploration. After full coding of all the focus group and interview transcripts, a set of key themes derived directly from the codes was generated. This involved listing all the descriptive codes, after which the researchers met to discuss their interpretation of these, combining or collapsing them accordingly to arrive at interpretative themes. These themes were then cross-checked and compared for verification and to generate thematically focused descriptive accounts of the groups and interviews. These provided a richer understanding.

## Results

### Overview

Participant characteristics are shown in Table 1. Practitioner work settings ranged from CYPMHS to student counseling to charity organizations, providing a rich variety of experiences. Young people were aged 16 to 24 years and self-reported a range of mental health diagnoses.

The results are presented separately for practitioners and young people. Themes identified for practitioners were (1) identifying priorities (asking young people about their web-based activities during clinical consultations); (2) practitioners’ knowledge, skills, and confidence in talking with young people about their web-based activities; and (3) the potential need for training and guidance. For young people, the key themes were (1) being left to cope (experiences of talking to practitioners about their web-based activities), (2) the impact of practitioners’ attitudes toward young people’s web-based activities, (3) trust and confidentiality in handling sensitive information, and (4) practitioners’ possible training needs. Finally, examples of good practice were obtained from both practitioners and young people.

**Table 1:** Participant characteristics (N=26).

		Young people (n=11)	Practitioners (n=15)
Age (years), median (range)		20 (16-24)	Not collected
**Gender, n (%)**			
	Man	0 (0)	4 (27)
	Woman	10 (91)	11 (73)
	Nonbinary	1 (9)	0 (0)
Country of origin and practice		United Kingdom	United Kingdom
**Ethnicity, n (%)**			
	White English	8 (73)	14 (93)
	White Scottish	1 (9)	0 (0)
	Black British	1 (9)	0 (0)
	Asian British	1 (9)	1 (7)
Years of practice, median (range)		N/A^a^	18 (5-43)
**Self-reported diagnoses, n (%)**			
	Anxiety and depression	2 (18)	N/A
	Depression	3 (27)	N/A
	Suicide or self-harm	1 (9)	N/A
	Eating disorder (anorexia nervosa)	4 (36)	N/A
	Borderline personality disorder	1 (9)	N/A
**Practitioner current role, n (%)**			
	CYPMHS^b^ consultant psychiatrist	N/A	3 (20)
	CYPMHS specialty physician	N/A	1 (7)
	CYPMHS psychiatry advanced training registrar	N/A	2 (13)
	Clinical lead, student services	N/A	1 (7)
	CYPMHS psychologist	N/A	1 (7)
	CYPMHS nurse	N/A	2 (13)
	Drug and alcohol worker	N/A	1 (7)
	CYPMHS family therapist	N/A	1 (7)
	University student well-being advisor	N/A	1 (7)
	Senior practitioner, third-sector organization	N/A	1 (7)
	Charity sector worker	N/A	1 (7)

^a^N/A: not applicable.

^b^CYPMHS: children and young people’s mental health services.

### Practitioners

#### Overview

In discussing the overall importance of having conversations about young people’s web-based activities, practitioners agreed that such conversations were important and should be a regular topic during clinical consultations:

I’m going to try a bit harder to try and encourage us to really make social media, the online world and online technology in general, a standard part of the session because I just don’t think that kids are really even considered, and yet for some it represents more than 50% of their social life.

CYPMHS family therapist

There was broad agreement among practitioners that the web-based world presented both harms and benefits to young people and that attention should focus on how to remain safe on the web (coping strategies) and how to access web-based support.

#### Identifying Priorities: Asking Young People About Their Web-Based Activities During Clinical Consultations

All practitioners except 1 (14/15, 93%) reported asking young people about their web-based activities during clinical consultations. Most emphasized the importance of exploring the emotional impact of the young person’s web-based experiences rather than asking about what digital technology or social media the young person used. Exploring how to manage risky web-based interactions was seen as valuable, and identifying harmful web-based behaviors to ensure risk management was routinely addressed in terms of young people’s safety. This included gaming addictions, Wii Fit use by individuals with eating disorders, accessing the dark web, problematic use of web-based dating apps, the dangers of web-based grooming, dealing with cyberbullying, and deciphering the appropriateness of a web-based friendship:

I will ask them how they spend their time, you know, what they do on the weekend...and normally then from there, some will come up with about gaming or about using their phone. It’s certainly not so important to me which programs, which applications so much as...what sort of things they’re looking at and why they’re doing that, how that connects to other things in their life.

Clinical lead; student services

There were 7% (1/15) of the participants who did not ask young people about their web-based activities unless it was mentioned and seemed not to make a potential connection between what the young person had seen on the web and their presenting problems:

Generally, when people have come in [it’s] because they’ve harmed themselves in some way or they’re in some kind of mental health crisis. So I’m mostly interested in what caused the self-harm and how it came about...so I wouldn’t specifically ask [about online activities] but if it comes up, then we talk about it.

CYPMHS nurse

Most variation arose in terms of to what extent, how frequently, and how conversations about web-based activities were conducted. Some discussed the topic routinely, but others checked just occasionally, often as part of a mental health assessment. A psychiatrist emphasized the importance of building a trusting relationship with the young person:

It’s no longer about telling them what to do...it’s about how you become a resource for them, and that you have a relationship with them that means that they trust you, that they come to you with the dilemmas that they’re in and share with you what they’re worried about.

CYPMHS psychiatrist

Others responded to young people’s negative experiences on the web by recommending that they come off social media or block certain people or sites:

It’s really difficult to get young people to switch off or to block. I don’t know if there’s a “fear of missing out” but it’s a lot to encompass all of it. We’ve been fighting with them because we want them to come offline and to start joining life that way.

Senior practitioner; third-sector organization

Another practitioner spoke of the positive aspects of social media in addition to the negative aspects, which had helped a young person with body image issues find support:

There’s one young girl, who is really struggling with an eating disorder and obviously there’s quite a lot of negativity online about pro-ana and that kind of thing online but actually, there’s a really big platform for body positivity at the moment. It’s just finding that balance between the negatives and the positives but it is the way we’re going and I feel restricting people and trying to get young people away from social media isn’t actually necessarily the way forward. It’s just about promoting more positives that come from it.

CYPMHS nurse

#### Practitioners’ Knowledge, Skills, and Confidence Talking With Young People About Their Web-Based Activities

Practitioners’ knowledge and skills about both the web-based world and young people’s web-based activities were gleaned largely from personal interest or informally in clinical settings (from colleagues and younger patients), as well as from family members. In total, 13% (2/15) of the practitioners (a charity sector worker and a consultant psychiatrist) had received training through their work. Having a “basic” knowledge of popular social media platforms, websites, and apps was seen as important, but having special expertise in the latest games, software, or web-based influencers was not considered necessary by all practitioners. Some described using their “ignorance” of the web-based world as a way of building rapport with a young person:

I don’t know any of the video games or anything, I mean I’ve really put my foot in it a lot of the times and pretended I know and then shown myself to be a complete idiot. I have no idea, but it means that the young person can explain it to me. So, I will say, “I have no idea how Twitter works. Tell me. What are you doing on there? How does that work?” And, you know, being the ignoramus that I am overtly allows them to tell me about it and that’s the way I do it.

Charity sector worker

Most practitioners (9/15, 60%) said that they felt confident in raising the topic of young people’s web-based activities during clinical consultations, but a few (4/15, 27%) did not:

I am not a user of social media, you know, I can WhatsApp but that’s basically it. So being one of the older members of staff I don’t feel that I am up to speed on what the latest things are or what to advise...you can maybe give advice for websites and things about medication or conditions or things like that. But I wouldn’t have used the apps as much.

CYPMHS psychiatry advanced training registrar

Those practitioners who felt limited because of their lack of knowledge recommended that the young person look elsewhere, for example, to a “specialist service”:

I would point them to a more specialist service, I don’t know, “Ask Frank” or something rather than delving into that because I would feel out of my depth, I would feel I would be doing them a disservice, whereas, yeah, if I had more knowledge, I could have that conversation.

Student well-being advisor

Practitioners who expressed a lack of confidence in exploring young people’s web-based activities said that the challenge was often in how to address the more extreme elements of the web-based world, such as sexual exploitation, pornography, radicalization, and the dark web:

I felt completely in the dark, sexual exploitation, it’s like organised crime now. I didn’t realise at the time how widespread it was...and I’d ask experienced colleagues what to do and how to kind of go about it (and) I was met with a lack of knowledge as well.

CYPMHS nurse

#### The Need for Training and Guidance

Many practitioners said that they wanted to increase their knowledge and confidence in communicating with young people about the web-based world, especially to update their understanding of recent developments in social media and mental health apps and understand the topic from a young person’s perspective:

Updates on—okay, this is what people are doing and this is what’s useful and this might be a problem, and just to be aware of that would be really good because it’s just changing all the time.

Charity sector worker

Some practitioners cited time constraints as a barrier to asking about young people’s web-based activities. This included having time during clinical consultations as well as time to acquire sufficient knowledge about the web-based world to be able to make useful recommendations and offer advice:

I mean there are a lot out there (apps) aren’t there, and we really don’t have time or the inclination to be looking through all the different mental health apps. We definitely don’t have time at work, but we don’t have the inclination when we’re not at work because we’re tired, and don’t want to think about it really. I think that’s the same for my colleagues as well. So I’ve got the ones I’ve used that I’ve found alright, so like Headspace, I would only ever recommend ones that I know. So I guess it would be useful to have somebody who’s already looked at them who’s a trusted source that says, “this one’s quite good,” you know.

CYPMHS nurse

### Young People

#### Overview

All the young people we spoke to (11/11, 100%) said that the web-based world presented both harms and positive elements in relation to mental health, emphasizing the web-based peer support that was available, especially through recognized sites such as Mind:

I think as well, like in the same way that it can be really toxic, you can find like really supportive communities...sometimes, you know, especially with depression if you’re having a bad day and then you’re scrolling through a lot of charities like Mind, they post a lot of uplifting quotes and stuff. And then you scroll through the comments and you see loads of other people that are saying, “I’m having a bad time at the moment,” it feels like that sense of community makes you feel like you’re not alone.

Focus group 2; aged 19 years

#### Being Left to Cope: Young People’s Experiences Talking to Practitioners About Their Web-Based Activities

Young people expressed a general desire to be able to talk to practitioners about concerns or harmful experiences they were having on the web and gain advice, but this was tempered by how comfortable they felt disclosing information to practitioners. A young participant said that it depended on the relationship with and experience of the practitioner:

I think it depends how down they are with this generation, how involved in the digital world they are themselves. There’re some health professionals who are just not very relatable to young people at all and so I’d say it really depends.

Focus group 1; aged 24 years

Young people reported that, although they wanted practitioners to have some understanding of digital technology and how young people use social media, they were more concerned about whether they felt comfortable disclosing their web-based activities to a practitioner in clinical consultations. Common barriers included feeling judged or misunderstood in terms of their web-based activities. Importantly, it was reported that the topic was seldom raised by practitioners. In fact, some young people reported having to raise the topic of their web-based activities themselves when this was concerning them rather than their practitioner doing so. Conversations that did occur could be superficial:

Well, I’ve never been like directly asked unless I’ve brought it up. So if I was like you know, “I’m really stressed, I’ve just seen this on Instagram” and then obviously that would open up the conversation about it, but I was never like directly asked.

Focus group 2; aged 19 years

I find that with all the mental health professionals that I’ve had, they don’t really ask about it and they don’t really, I don’t know, it might come up in conversation. It’s just, it’s never really something that is gone into in too much depth. I don’t really think they focus on that [...] and I just think maybe their training doesn’t include things about digital technology and stuff like that. Which I think given this day and age, I really think that would be relevant for them to have certain training on talking about that.

Focus group 1; aged 20 years

Conversations that were focused on the young person coming off social media or blocking certain people in response to reporting negative web-based experiences were said to compound feelings of isolation and loneliness. This also led to the blocking of content that was supportive, thereby preventing potentially useful conversations on how to manage web-based harms:

I think loneliness is something as well because when you have social media you can connect to people and it’s sort of like, if you didn’t have access to that who knows you might be really lonely. A lot of people talk about mental health online as well, a lot of influencers. And there’s been times of my life where I’ve not had my phone, like I’ve been in care or in hospital and my phone’s been taken away from me, but with them taking it away from me they forced me to really spiral into my depression even more.

Focus group 1; aged 17 years

So I think saying like, you know, you’re in crisis so you can’t use social media, that’s not really going to solve anything. Because if someone is in crisis regardless of what their risk is online or offline, people will find a way. So by blocking social media that doesn’t solve the problem or cause the problem. [...] And it’s about learning to manage it healthily.

Focus group 2; aged 22 years

Lack of conversation with a practitioner about harmful web-based activities meant that young people felt left to cope and manage problems on their own rather than having an informed conversation about coping strategies:

Yeah, I think we’ve got to talk about it [harmful online content] a little bit at least, so if we could perhaps talk about that and replace it with maybe some better coping techniques. Also yeah, it would be nice to feel like that was an area that you could talk about, (but) it’s just absolutely ignored. So if there was stuff that came up, yeah it would be nice to feel like that’s normal to bring up.

Focus group 3; aged 18 years

#### The Impact of Practitioners’ Attitudes Toward Young People’s Web-Based Activities

Although suggestions to block or come off social media were considered unhelpful, so were attitudes that indicated a lack of response or interest, meaning that it was a struggle for some young people to ask for help when encountering harmful web-based content:

I had a bad experience of trying to tell people like, “this is really hard for me, like really, really hard,” like actually trying to tell someone. And then I told them [practitioner] and nothing happened and it was just really draining, like I just kept like I kept telling them and it was just that like nothing ever happened. So when I finally did get on to someone I just felt like everybody was really repetitive, I never felt like there was anything personal, it just felt like routine, like “whatever.”

Focus group 2; aged 21 years

This feeling of being dismissed could also occur when the young person felt that they were “fobbed off” with apps that seemed out-of-date:

If they had training about this they might then be able to really advise you on some of the things that may help. Now in the past like my psychologist she recommended a bunch of apps [...] And a lot of these apps they’re not on par with everything else, they seem a bit more like, old fashioned.

Focus group 1; aged 18 years

Focus group participants discussed how stigmatization of the web-based world by practitioners led to them feeling that they were held responsible or culpable for what happened to them on the web and were “not trying hard enough” to protect themselves on the web. This was reported alongside a lack of recognition of the fact that young people are routinely exposed to harmful content on the web without having to proactively search for it:

I think sometimes from that practitioner, [it’s] like a blaming of young people, or I feel like it met with the response of, “you’re not trying hard enough to get better” when actually it wasn’t as if I was consciously looking for these images or whatever. Sometimes it just kind of comes up or whatever. And I think there is quite a stigma about social media and things.

Focus group 2; aged 19 years

It was like, glossed over. The question was pretty much, “Are you making sure that you’re avoiding your triggers?” in a nicer way. And so they were really holding me to be accountable when actually, no, I wasn’t because it’s so easy to find them [triggers] online.

Focus group 1; aged 20 years

#### Trust and Confidentiality in Handling Sensitive Information

A key aspect of whether a conversation took place or was helpful was having trust in a practitioner’s skills in handling sensitive information, including what happened to the information shared during sessions and how web-based risk was dealt with. For example, a focus group member said that her parents were informed without her knowledge about an unsafe site she had used, which led to the removal of all her devices for 2 years and, consequently, a lack of any conversation about how to keep safe on the web:

I was under CAMHS (and) I had a conversation with the CAMHS worker about like an unsafe website that I was using. And they broke confidentiality to my parents about that, without informing me, which is a whole other issue. But basically, what led to that was then my parents basically blocking all websites so I couldn’t access any websites at school for about like a year or two.

Focus group 1; aged 23 years

Trust also depended on the practitioner demonstrating some of their personal experiences of the web-based world in relation to knowledge of appropriate mental health apps:

Yeah, I think it’s having that trust in the professionals that they know what they’re on about, [...] that they’ve got experience in whatever it is they’re saying or telling you to do. That builds a level of trust as well, it’s not just them giving you some advice they’ve read. They’ve got that experience maybe.

Focus group 2; aged 21 years

#### Practitioner Training Needs

Young people agreed that there was a need for guidance or training on how practitioners could address young people’s mental health related to their web-based activities:

I think that professionals talking about your online usage and digital, just all of that in general, might be a little bit pointless and redundant unless there is a lot of [training]. I think it can just be very pointless if they don’t know what they’re talking about.

Focus group 1; aged 20 years

They thought that young people themselves should be involved in such training, either by contributing their own experiences through discussions with practitioners or by providing information about how young people view and use digital technology:

I do think that with the training, I think it’s vital that young people are the heart of that, or at least have a big role in that...Because the age of technology, you know, it’s not been around for a very long time and we’ve sort of grown up with it. Whereas a lot of the professionals now, they haven’t...So they might not understand it in the same way that we do.

Focus group 2; aged 22 years

However, a general view among young people suggested that things were changing for the better, which was thought to be because of the presence of younger staff who were more adept in and familiar with the web-based world:

I guess it helps that there’s like all these new cohorts of student nurses for example coming in who have that experience as well, like using social media or using the internet and I think it’s becoming a lot more widely accepted that that’s the way that people can access help or support and I think it is getting a lot better.

Focus group 2; aged 22 years

#### Examples of Good Practice

A young person cited a positive experience she had with a practitioner whereby they looked together at some of the harmful sites she had visited and then discussed suitable alternatives:

We just sat down and she just asked me about like my social media use, any apps that I had that were not necessarily the best apps to have, which is how I became aware of the toxic-ness of like my activity apps and stuff which for whatever reason I just hadn’t considered. And so it was through that, she just directly asked me basically...[and] she was like very straight-talking and honest and I appreciate that. [...] It was a professional saying, “I used this and I found it really useful and I’m recommending it to you as a result.”

Focus group 2; aged 21 years

One of the more experienced practitioners used apps regularly during sessions and discussed coping strategies with young people to deal with harmful content. This included support to remove a gambling app and block access during certain periods, which helped manage a gambling addiction:

It might be the focus of treatment so if someone is trying to reduce their problematic gambling, part of it might be removing the XX app, or it might be thinking, actually can we put a block on when you can use the app or can we only use the app for certain occasions...so again, thinking a bit about how that gambling cycle’s broken. Or making people aware of things like, for example, Loot boxes and microtransactions in video games [that] are more or less gambling. People don’t see it as spending lots of money, so you might actually have a conversation around, “you do realise what this is? this is how a Loot box works.”

Clinical lead; student services

## Discussion

### Principal Findings

Social media continues to pose a challenge to young people’s mental health, as highlighted by young participants in this study; this includes harms that are not always easily identifiable [6]. The findings indicate that practitioners are potentially in a good position to provide this space and help young people identify the harms and positive aspects of their use of social media. As identified in the literature, there are also considerable benefits for young people in using social media if they feel confident in discussing their experiences and are able to ask how to access useful and constructive support as well as how to interact safely with others on the web [17,18,25].

We found broad agreement between young people and mental health practitioners on the fact that young people’s web-based activities should be discussed during clinical consultations. Young people thought that such conversations mostly were dismissed or glossed over or did not take place at all. Advice from practitioners that young people should stop their web-based activities or using devices—without either clear reasons for doing so or the agreement of the young person—was considered unhelpful and meant that young people felt stigmatized or judged when encountering negative content on the web.

We also found that there was general agreement among young people about what the potential content of their interactions with practitioners about web-based activity could be, for instance, the opportunity to receive advice about ways to manage harmful web-based content, learn coping strategies, and explore reasons for engagement with certain types of harmful web-based activities and how this affects mental health. Young people said that they would value discussion and advice from practitioners based on their experience that are relevant to the young person, for example, which apps are suitable and which apps to avoid.

Although practitioners expressed confidence in talking to young people about their mental health related to their web-based activities, there was also a clear desire to learn more about the web-based world and how to assist young people in navigating their way through it as safely as possible. This learning would provide an understanding of web-based activities from young people’s viewpoints, which would enable discussions about the use of digital technology, and especially social media, from a position of knowledge and inclusivity. Practitioners who had more experience talking with young people about their web-based activities shared how conversations could have the most impact, namely, displaying awareness of some of the latest social media platforms and suitable apps.

Despite recommendations cited in the literature [11,13] stating that consultations between health care professionals and young people should include conversations about social media and its impact on young people’s mental health, this is the first qualitative study to capture the experiences of both groups in talking about this topic during clinical consultations. Both groups in our study identified a need to include young people in the creation and delivery of training and guidance for practitioners to begin tackling some of these issues.

Our findings indicate that such conversations currently do not always meet the needs of young people, who lack assurance that practitioners have a sufficient understanding of the impact of the web-based world on young people’s lives or how they engage with it to provide sensitive or realistic responses. Such understanding was revealed as fundamental to trust and willingness to disclose. Our findings also imply that “how” practitioners ask young people about their web-based activities may be as important as “what” they ask. To this end, our further work has focused on establishing young people and practitioner good practice guidance [21].

### Strengths and Limitations

Practitioners who took part were self-selected and likely to have done so because of interest in the topic. This means that the views included in the study may not fully reflect those of the wider population of mental health practitioners. Similarly, the young people also self-selected, and their views might be different from those of the wider youth population. Despite efforts to include all genders in the young people sample, we were unable to recruit any men. This absence is a considerable limitation as we are aware that their experiences and views may be different from those of others. An anonymous survey was conducted before the qualitative component that also showed a gender imbalance; there were fewer male respondents than female respondents, suggesting that male individuals are a harder-to-reach group that may require a different approach in recruiting for research. Future research may wish to consider other ways of collecting information that enable greater inclusion in research, for instance, as with the practitioners, the option of an individual interview.

In each subgroup, the sample size was guided by the principle of information power to ensure an adequate sample size [26], namely, that recruitment efforts continued until a sufficient sample size was achieved. The sample sizes for all parts of the study were adequate and appropriate for a study of this type where the objective was an in-depth exploration of the topic that would allow for probing and follow-up questions that would assist in obtaining a deeper perspective. Participants from both samples offered specificity in terms of their expertise either as mental health practitioners or as young people with lived experience. Therefore, the study aims were fully addressed through the richness and depth of participants’ knowledge and experience. The method of analysis further adds to our confidence that the findings are robust and answer the study aims through the process of double coding that ensured reliability by combining both focus groups and interviews. The findings may be transferable to other study populations such as adult mental health services and school settings.

### Conclusions

Mental health practitioners may be in a unique position to assist young people with mental health problems in navigating the various challenges and opportunities that the web-based world presents. Young people in our study expressed a willingness to learn safer ways to navigate the web-based world and explore why they engage in harmful web-based behavior; however, they seldom initiated or shared their experiences with practitioners out of fear of being judged. Opportunities for meaningful and constructive conversations are being missed, but this could be resolved with increased guidance for practitioners on how to approach and support young people’s safer use of social media and digital technology [21,27]. A degree of knowledge exchange between young people and practitioners will be essential, and both groups expressed a desire for any training or guidance to be co-designed between practitioners and young people. Key challenges will be to address practitioner concerns about time constraints and the feasibility of regularly asking about web-based activities and identify methods for keeping practitioners abreast of rapidly changing social media trends.

## Abbreviations

CYPMHS: children and young people’s mental health services

## Data Availability

The data sets generated and analyzed during this study are available from the corresponding author upon reasonable request.
